# LincRNA FEZF1-AS1 contributes to the proliferation of LAD cells by silencing p57 expression

**DOI:** 10.18632/oncotarget.21265

**Published:** 2017-09-23

**Authors:** Shuai Jin, Siyu Chen, Yongfu Ma, Bo Yang, Yang Liu

**Affiliations:** ^1^ Department of Thoracic surgery, PLA General Hospital, Beijing, 100853 China

**Keywords:** lung adenocarcinoma, FEZF1-AS1, LSD1, EZH2, H3K4me2

## Abstract

LincRNA FEZF1-AS1 has been identified to exert oncogenic functions in various biological processes of tumorigenesis. However, the function of FEZF1-AS1 in lung adenocarcinoma still remains unclear. Our findings revealed that FEZF1-AS1 was increased in lung adenocarcinoma tissues and cell lines and high level of FEZF1-AS1 was associated with poor prognosis of lung adenocarcinoma. Functional experiments and mechanistic investigations demonstrated that knockdown of FEZF1-AS1 significantly repressed proliferation through influencing the distribution of cell cycle. Besides, we also uncovered that FEZF1-AS1 could suppress p57 expression through recruiting EZH2 and LSD1 to the promoter of p57, thus influenced the cell cycle and proliferation. Collectively, our results suggested that FEZF1-AS1 was involved in the progression of lung adenocarcinoma and might be as a potential therapy target for human lung adenocarcinoma.

## INTRODUCTION

Lung adenocarcinoma (LAD), as the commonest histological type of non-small cell lung cancer (NSCLC), has been identified as the leading cause of cancer-related deaths worldwide [1]. Despite many efforts have been made recently, the prognosis of LAD still remains unsatisfied. Since LAD involves the dyregulation of oncogenes and tumor suppressers, it is essential for early diagnosis and efficient treatment to further investigate the initiation and progression of its underlying mechanism.

Long non-coding RNAs (lncRNAs), with more than 200nt and limited or no protein-coding capacity [2, 3], have been identified dysregulated in many human diseases, including tumorigenesis [4–6]. For example, H19/let-7/LIN28 reciprocal negative regulatory circuit promotes breast cancer stem cell maintenance [7]. HOTAIR promotes metastasis of renal cell carcinoma by up-regulating histone H3K27 demethylase JMJD3 [8]. MEG3 contributes to nickel malignant transformation of human bronchial epithelial cells via modulating PHLPP1 transcription and HIF-1α translation [9]. FEZ family zinc finger 1 antisense RNA 1(FEZF1-AS1), locating at the opposite strand of FEZF1 gene, has been recently identified as a long non-coding RNA and its dyregulation has been reported in gastric cancer and colorectal cancer [10, 11]. However, its function in LAD remains unknown.

In our present study, we measured the expression level of FEZF1-AS1 in LAD tissues, matched adjacent normal tissues and LAD cell lines, and analyzed its correlation with clinicopathological factors of patients. Functional experiments were performed to measure the biological effect of FEZF1-AS1 and mechanistic studies were employed to investigate the associated between EZH2, LSD1 and FEZF1-AS1. The results from the experiments proved that the epigenetic repression of p57, which might be at least partially responsible for FEZF1-AS1-mediated proliferation in LAD.

## RESULTS

### FEZF1-AS1 is upregulated in LAD and is correlated with TNM stage

**Figure 1:**
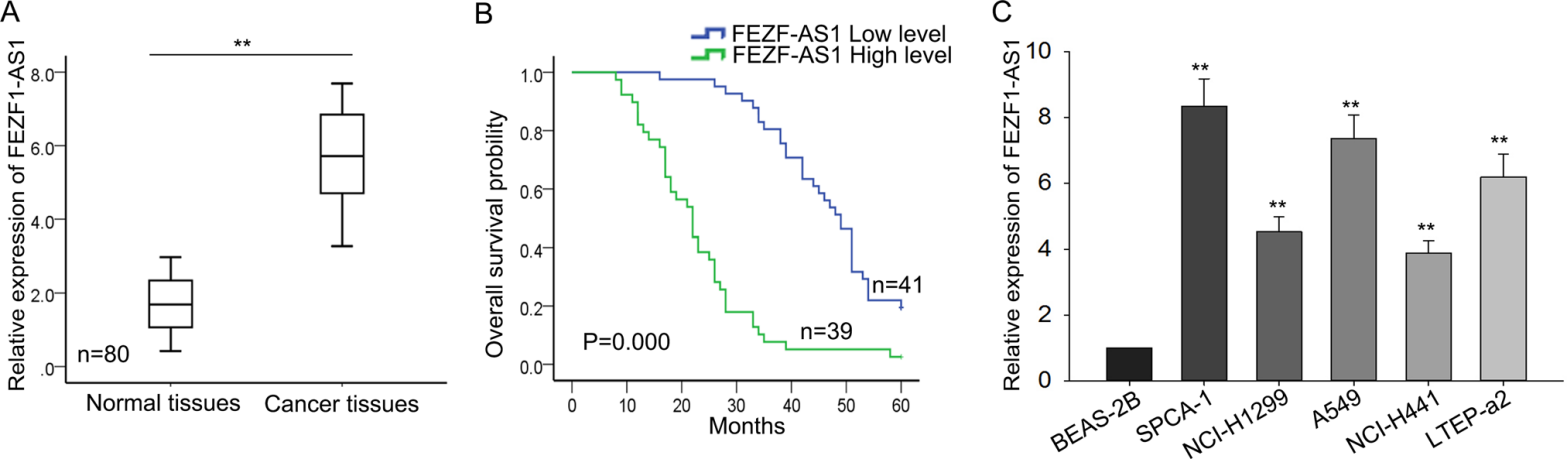
FEZF1-AS1 is upregulated in LAD and is significantly correlated with TNM stage (**A**) The level of FEZF1-AS1 in LAD tissues was measured by qRT-PCR. (**B**) Kaplan-Meier method analysis (log-rank test) was employed to analyze the correlation between FEZF1-AS1 level and overall survival of patients. (**C**) The level of FEZF1-AS1 in LAD cell lines was detected by qRT-PCR. ^*^P < 0.05, ^**^P < 0.01, Means ± SD. were shown.

To explore the biological function of FEZF1-AS1 in LAD, we measured the expression level of FEZF1-AS1 in 80 pairs of LAD tissues and corresponding normal tissues. As shown in Figure 1A, FEZF1-AS1 expression was obviously more increased in the LAD tissues than in the corresponding normal tissues (*P* < 0.01). To evaluate whether FEZF1-AS1 expression was correlated with clinical pathological parameters and prognosis of LAD, 80 patients were divided into FEZF1-AS1 highly expression group (*n* = 39) and FEZF1-AS1 lowly expression group (*n* = 41) according to the cutoff value which was based on the mean expression level of FEZF1-AS1. As observed in Table 1, high level of FEZF1-AS1 was significantly correlated with tumor size (*P* = 0.003), TNM stage (*P* = 0.007) and lymph node metastasis (*P* = 0.001), rather than with age, gender, smoking and differentiation (*P* > 0.5). Furthermore, Kaplan–Meier method analysis (log-rank test) was employed to analyze the correlation between the FEZF1-AS1 expression and overall survival of patients which revealed that high level of FEZF1-AS1was associated with poor prognosis (*P* = 0.000, log-rank test; Figure 1B). Proportional hazards method analysis revealed that high level of FEZF1-AS1 could be acted as prognostic factor (Table 2, *P* = 0.001). Additionally, we assessed the endogenous expression level of FEZF1-AS1 in five LAD cell lines (SPCA-1, NCI-H1299, A549, NCI-H441, LTEP-a2) and a normal lung epithelial cells BEAS-2B. As obtained in Figure 1C, expression level of FEZF1-AS1 was obviously increased in five LAD cell lines, among which its level in A549 and SPC-A1 was highest. Therefore, we chose A549 and SPC-A1 for the loss of function assays. Collectively, these findings suggested that FEZF1-AS1 might act as an oncogene in LAD progression.

**Table 1:** Correlation between FEZF1-AS1 expression and clinical features (*n* = 80)

Variable	FEZF1-AS1 Expression		*P*-value
	low	high	
**Age**			
≤ 65	21	16	0.380
> 65	20	23	
**Gender**			
Male	25	26	0.647
Female	16	13	
**Smoking**			
Smoking	18	19	0.823
No smoking	23	20	
**Differentiation**			
Poor	20	25	0.184
Well/Moderate	21	14	
**Tumor size**			
≤ 3 cm	30	15	0.003^*^
> 3 cm	11	24	
**TNM stage**			
I–II	29	15	0.007^*^
IIIa	12	24	
**Lymph metastasis**			
Absent	29	12	0.001^*^
Present	12	27	

Low/high by the sample mean. Pearson χ^2^ test. *P* < 0.05 was considered statistically significant.

**Table 2:** Multivariate analysis of prognostic parameters in patients with LAD by Cox regression analysis

Variable	Category	*P*-value
**Age**	≤ 60	0.603
	> 60	
**Gender**	Male	0.475
	Female	
**Smoking**	Smoking	0.572
	No smoking	
**Differentiation**	Poor	0.463
	Well/Moderate	
**Tumor size**	≤ 3cm	0.013^*^
	> 3cm	
**TNM stage**	I–II	0.785
	IIIa	
**Lymph metastasis**	Absent	0.397
	Present	
**FEZF1-AS1 expression**	Low	0.001^*^
	High	

Proportional hazards method analysis showed a positive, independent prognostic importance of FEZF1-AS1 expression (*P* = 0.001). ^*^P < 0.05 was considered statistically significant.

### Silenced FEZF1-AS1 suppressed LAD cells proliferation

**Figure 2:**
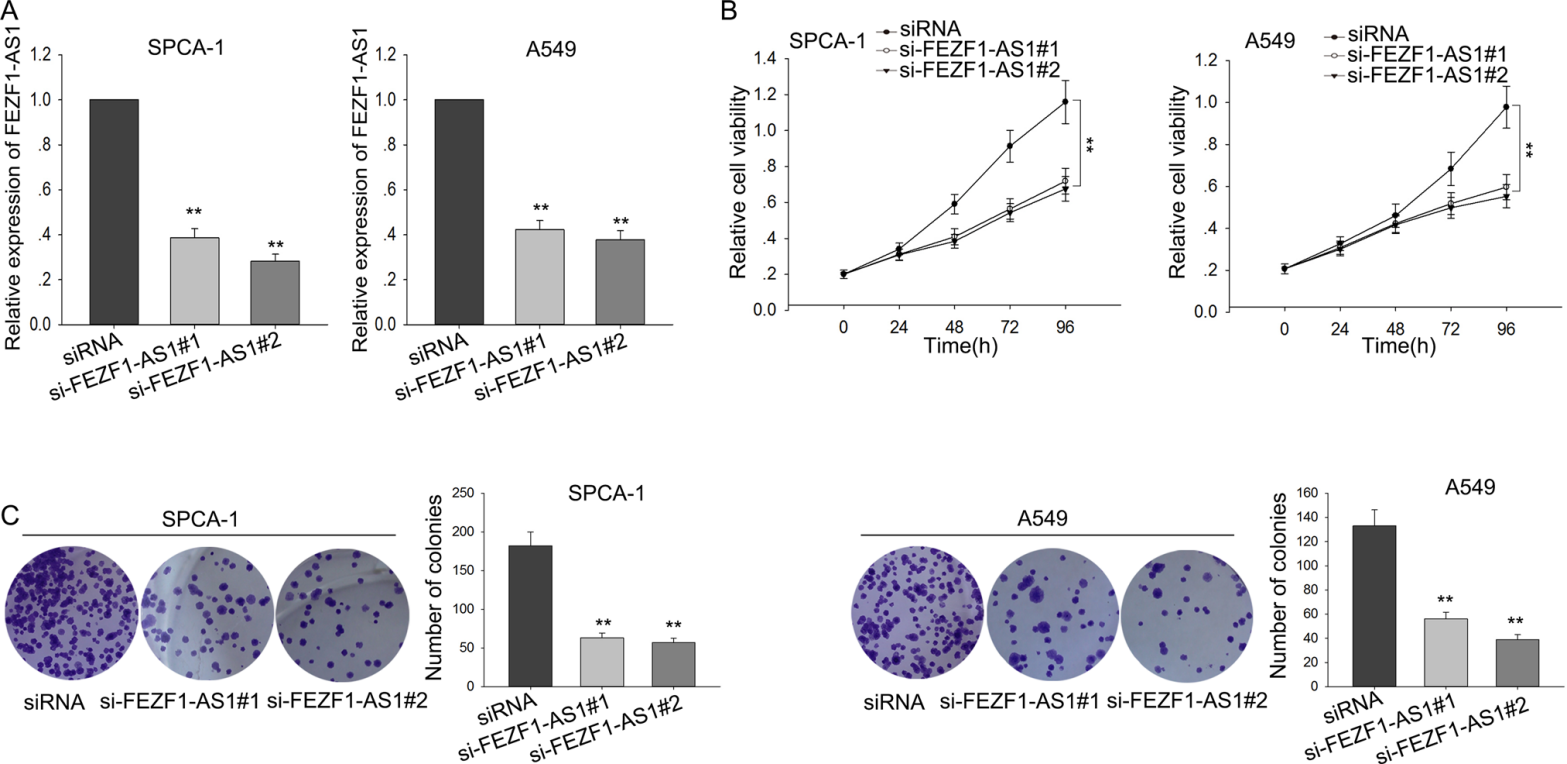
Silenced FEZF1-AS1 suppressed LAD cells proliferation (**A**) A549 and SPC-A1 cells were transfected with si-FEZF1-AS1 and transfection efficiency was measured by qRT-PCR. (**B**–**C**) The effect of FEZF1-AS1 knockdown on cell proliferation was measured by MTT and colony formation assays. ^*^P < 0.05, ^**^P < 0.01, Means ± SD. were shown.

To investigate the biological function of FEZF1-AS1 in LAD cells, A549 and SPC-A1 cells were transfected with specific siRNA (si-FEZF1-AS1#1 and si-FEZF1-AS1#2) to silence the endogenous FEZF1-AS1, and transfection efficiency was obtained after 48h (Figure 2A). Then, MTT assays were applied and the results showed that silenced FEZF1-AS1 significantly reduced the cell viability, compared with the respective controls (Figure 2B). Consistent with the MTT, colony formation assays also revealed that cells clonogenic survival was obviously suppressed when FEZF1-AS1 was knockdown (Figure 2C). Taken together, these results indicated that FEZF1-AS1 promoted the tumorigenicity and growth of LAD.

### Changes from cell cycle and apoptosis contribute to the functions of FEZF1-AS1

**Figure 3:**
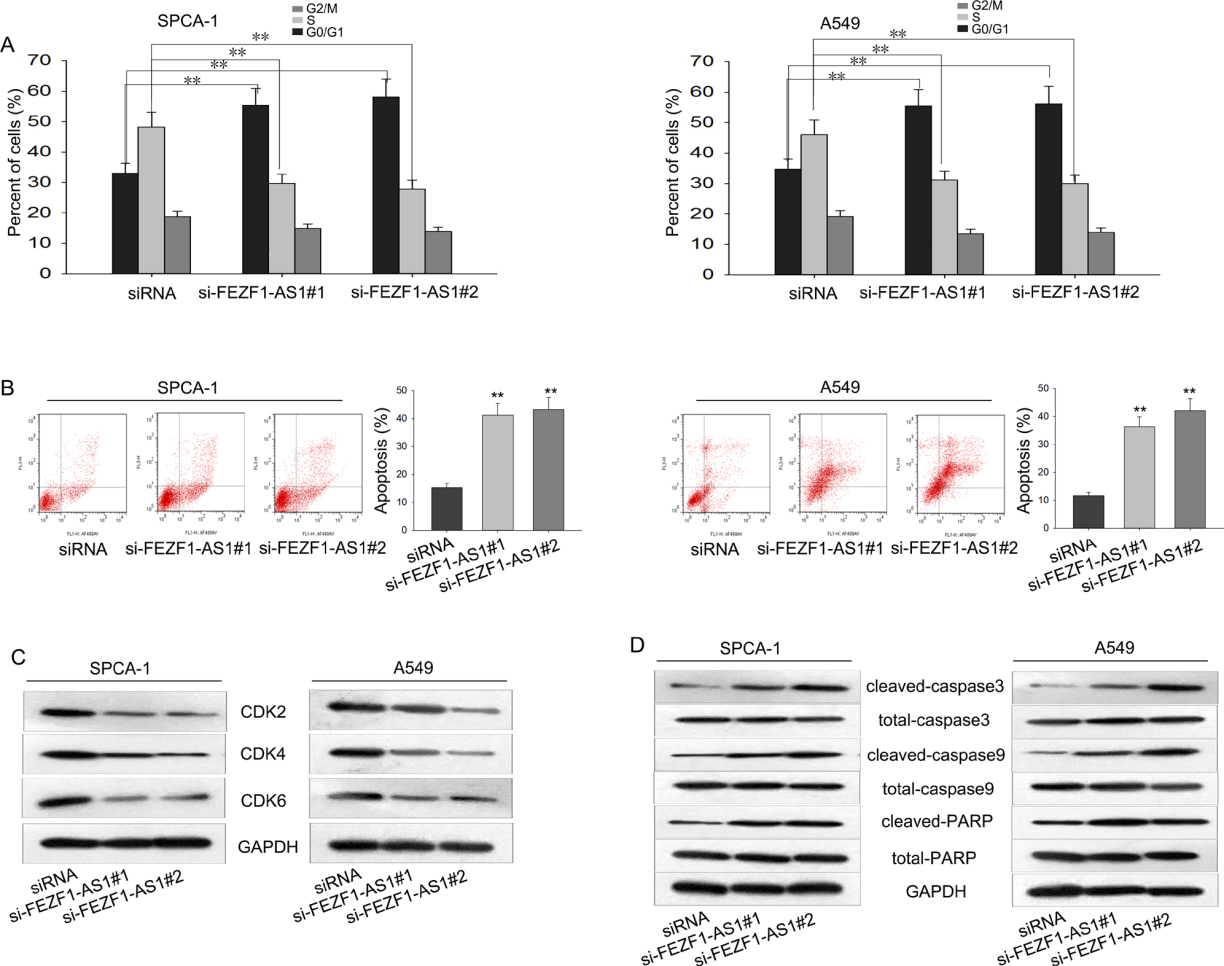
Changes from cell cycle and apoptosis contribute to the functions of FEZF1-AS1 (**A**–**B**) The effect of silenced FEZF1-AS1 on cell cycle and apoptosis was analyzed by flow cytometric analysis. (**C**–**D**) Western blot was performed to determine the levels of CyclinD1/CDK2/CDK4/CDK6 and apoptosis-related proteins. ^*^P < 0.05, ^**^P < 0.01, Means ± SD. were shown.

The dysregulation of cell cycle was responsible for tumor proliferation. Therefore, we hypothesized that whether the growth-inhibition effect caused by silenced FEZF1-AS1 was through affecting cell cycle and cell apoptosis. To prove the hypothesis, flow cytometric analysis of cell cycle was utilized and the results revealed that knockdown of FEZF1-AS1 significantly caused cell cycle arrest at G1 (Figure 3A). Then, flow cytometric analysis of apoptosis was performed to detect the function of FEZF1-AS1 on apoptosis in LAD cells. As presented in Figure 3B, deletion of FEZF1-AS1 significantly increased the apoptosis rate of A549 and SPC-A1 cells. These data indicated that FEZF1-AS1 contributed to the proliferation ability of LAD cells due to influence on cell cycle and apoptosis. Additionally, western blot was performed to measure the protein levels of CDK2/CDK4/CDK6 and apoptosis related proteins in the cells transfected with si-FEZF1-AS1. As shown in Figure 3C and 3D, the protein levels of CDK2/CDK4/CDK6 were obviously reduced and apoptosis-related proteins cleaved-caspase3, total-caspase3, cleaved-caspase9, total-caspase9, cleaved-PARP and total-PARP were significantly increased in A549 and SPC-A1 cells when FEZF1-AS1 was silenced. These results confirmed that FEZF1-AS1 caused cell-cycle arrest and induced apoptosis.

### FEZF1-AS1 epigenetically silenced p57 by recruiting EZH2 and LSD1 to the promoter of p57

**Figure 4:**
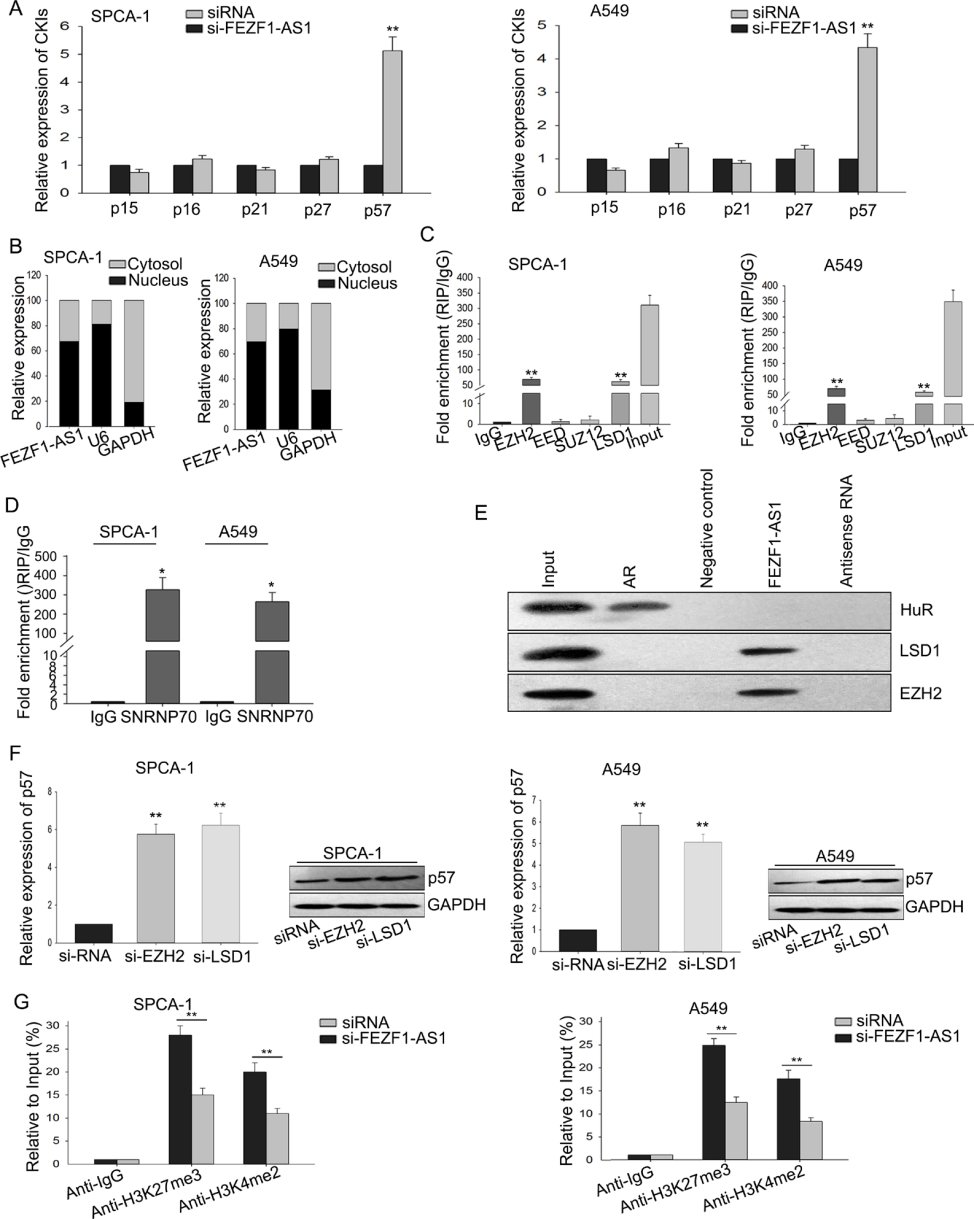
FEZF1-AS1 epigenetically silenced p57 by recruiting EZH2 and LSD1 to the promoter of p57 (**A**) The levels of CKIs were measured by qRT-PCR in A549 and SPC-A1 cells when FEZF1-AS1 was knockdown. (**B**) The level of FEZF1-AS1 in nucleus versus cytosol was measured by qRT-PCR. (**C**) RIP assay was performed by using EZH2, LSD1 antibodies for immunoprecipitation. (**D**) U1 binding with SMPNR70 was used as a positive control. (**E**) Biotinylated FEZF1-AS1 or antisense RNA was incubated with total-cell extracts (SPC-A1 cells); the level of EZH2 and LSD1 proteins in immunoprecipitates with FEZF1-AS1 was measured by western blot assay. The immunoprecipitates of HuR protein with AR RNA was used as a positive control. (**F**) p57 mRNA and protein levels were detected by qRT-PCR and western blot assay when FEZF1-AS1 was knockdown. (**G**) ChIP-qRT-PCR of EZH2 occupancy and H3K27me3 or LSD1 occupancy and H3K4me2 binding to the promoter of p57 in A549 and SPC-A1 cells were treated with si-FEZF1-AS1 or si-RNA; IgG was a negative control. ^*^P < 0.05, ^**^P < 0.01, Means ± SD, were shown.

To confirm the function of FEZF1-AS1 on cell cycle, we evaluated the expression level of CDK inhibitors (CKIs) (p15, p16, p21, p27, p57). As shown in Figure 4A, silenced FEZF1-AS1 significantly increased the level of p57, compared with control group. p57 is an inhibitor for cyclin-dependent kinase, and is deemed as a candidate of tumor-suppressive gene that has been identified dysregulated in numerous of cancers [12–15]. In addition, p57 is also a direct target of EZH2 and repressed by several epigenetic mechanisms in ovarian cancer, breast cancer and non-small cell lung cancer [16–18]. It demonstrated that FEZF1-AS1 epigenetically repress p21 through LSD1-mediated H3K4me2 demethylation [10]. To further explore the mechanism of FEZF1-AS1 in regulating cell cycle, we assessed the level of FEZF1-AS1 in nucleus versus cytosol by qRT-PCR. As illustrated in Figure 4B, the level of FEZF1-AS1 was obviously higher in nucleus than cytosol, indicating that FEZF1-AS1 exerted its function in LAD possibly at the transcriptional level. Therefore, we hypothesized that the function of FEZF1-AS1 in LAD might be mediated by LSD1 and EZH2. Next, we performed RNA immunoprecipitation assays to confirm that FEZF1-AS1 could bind directly to EZH2 and LSD1 in LAD cells. As shown in Figure 4C, we observed that endogenous FEZF1-AS1 was significantly enriched in the anti-LSD1 and anti-EZH2 RNA-IP fraction, both in A549 and SPC-A1 cells, and U1 binding with SNRNP70 was used as a positive control (Figure 4D). Furthermore, RNA pull-down assays further confirmed that FEZF1-AS1 indeed binds bound with to EZH2 and LSD1 in LAD cells (Figure 4E). Next, to ensure that FEZF1-AS1 inhibited p57 through the mediation of LSD1 and EZH2, we down-regulated LSD1 and EZH2 respectively in LAD cells with LSD1-specific and EZH2-specific siRNAs, and then measured the level of p57. As presented in Figure 4F, the level of p57 was significantly increased when LSD1 or EZH2 was down-regulated. We next performed chromatin immunoprecipitation assays and found that the knockdown of FEZF1-AS1 suppressed their binding ability and induced modification (Figure 4G). The above results suggested that FEZF1-AS1 regulated p57 in LAD cells mainly through recruiting RNA binding proteins EZH2 and LSD1 to the promoter regions of p57 and inducing histone modification.

### The oncogenic function of FEZF1-AS1 was partly dependent on the repression of p57

**Figure 5:**
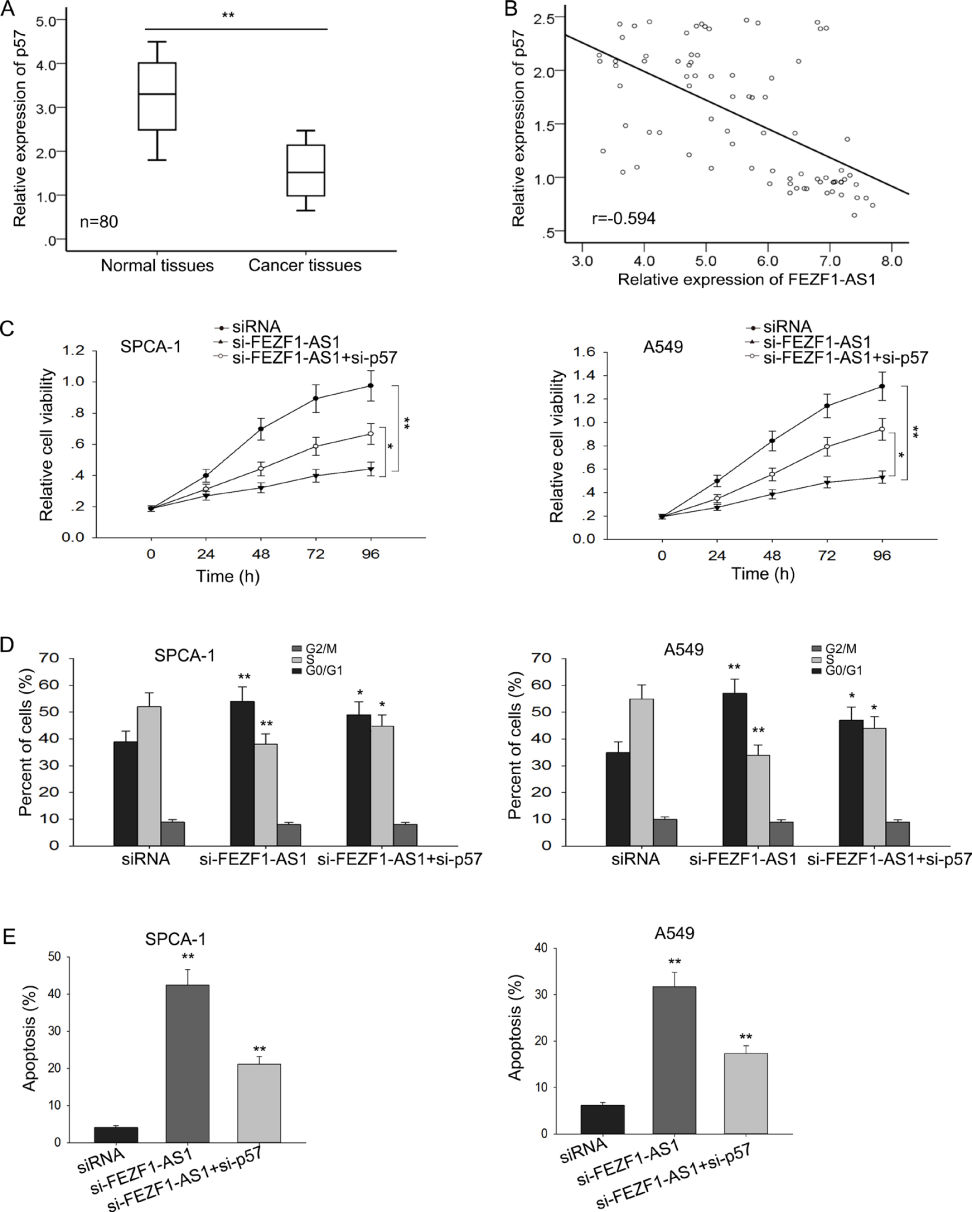
The oncogenic function of FEZF1-AS1 was partly dependent on the repression of p57 (**A**) The level of p57 in LAD tissues and corresponding normal tissues was measured by qRT-PCR. (**B**) The correlation between p57 and FEZF1-AS1 was analyzed. (**C**) MTT assay were performed to determine the proliferation ability of LAD cells transfected with siRNA, si-FEZF1-AS1 or si-p57. (**D**–**E**) Flow cytomery analyses were performed to analyze the cell cycle distribution and apoptosis rate of LAD cells transfected with siRNA, si-FEZF1-AS1 or si-p57. ^*^P < 0.05, ^**^P < 0.01, Means ± SD, were shown.

To further ascertain whether p57 mediated the function of FEZF1-AS1 in LAD, we firstly detected their expression levels in LAD tissues and corresponding normal tissues and analyzed the correlation between p57 and FEZF1-AS1. As shown in Figure 5A, the expression of p57 was significantly decreased in LAD tissues, and was negative correlated with the expression level of FEZF1-AS1 (Figure 5B). Then, we performed rescue experiments. A549 and SPC-A1 cells were co-transfected with si-FEZF1-AS1 and si-p57. Results from MTT showed that knockdown of p57 could partly abolish the si-FEZF1-AS1-mediated growth inhibition in A549 and SPC-A1 cells (Figure 5C). Moreover, cell cycle arrest and increased apoptosis rate caused by silenced FEZF1-AS1could be partially rescued by the introduction of si-p57 in A549 and SPC-A1 cells (Figure 5D–5E). Collectively, these results demonstrate that FEZF1-AS1 acted as an oncogene in human LAD through targeting p57.

## DISCUSSION

Currently, due to their critical function in tumorigenesis and cancer progression, lncRNAs have been attracting more and more attention from researchers. For instance, Cao et al. [19] revealed that downregulation of lncRNA CASC2 by microRNA-21 increased the proliferation and migration of renal cell carcinoma cells; Zhang et al. [20] illustrated that Antisense lncRNA FOXC2-AS1 promoted doxorubicin resistance in osteosarcoma by increasing the expression of FOXC2; and Liu et al. [21] related that epigenetic silencing of miR-218 by the lncRNA CCAT1, acting via BMI1, promoted an altered cell cycle transition in the malignant transformation of HBE cells induced by cigarette smoke extract. Although so many lncRNAs have been studied, the potential biological functions and molecular mechanistic details for most lncRNAs in human LAD still remain to be investigated. FEZF1-AS1, an lncRNA producing a 2564 bp transcript, and located in chromosome 7A, is significantly associated with the progression of gastric cancer colorectal carcinoma [10, 11]. In our present study, we uncovered that FEZF1-AS1 was significantly overexpressed in LAD tissues and cells. High level of FEZF1-AS1 was associated with poor prognosis in patients with LAD. Functional experiments revealed that FEZF1-AS1 boosted LAD cell proliferation through influencing cell cycle and apoptosis rate. Silenced FEZF1-AS1 obviously suppressed LAD cell growth, induced apoptosis, and caused cell cycle arrest. These findings indicated that FEZF1-AS1 functioned as an oncogene in LAD, and its overexpression accelerated the progression and development of LAD.

Generally, lncRNAs regulate the expression of target genes via multiple mechanisms such as genomic imprinting, chromatin modification, RNA decay and act as ceRNAs sponging miRNAs [22–24]. And many studies have shown that lncRNAs regulate target expression through distinct mechanisms in different cancer cells which are partially dependent on their locations [18, 25]. To study the mechanism by which FEZF1-AS1 exerted its function in LAD, we determined the expression level of FEZF1-AS1 in LAD cells and found that FEZF1-AS1 was mainly located in nucleus, indicating that FEZF1-AS1 regulated target gene probably at transcriptional level. Furthermore, with RNA immunoprecipitation (RIP) and RNA pull-down analyses, we showed that FEZF1-AS1 could bind directly to RNA-binding proteins EZH2 and LSD1. p57, as an inhibitor for cyclin-dependent kinase, has been deemed as a candidate of tumor-suppressive gene and dysregulated in numerous of cancers [12–15]. In addition, p57 is also a direct target of EZH2 and repressed by serveral epigenetic mechanisms in ovarian cancer, breast cancer and non-small cell lung cancer [16–18]. And our findings revealed that silenced FEZF1-AS1 could markedly increase the level of p57 while knockdown of EZH2 or LSD1 also obviously increased the expression level of p57 in LAD cells, suggesting that FEZF1-AS1 might regulate p57 through interacting with EZH2 and LSD2. Additionally, ChIP assays determined that FEZF1-AS1 could simultaneously recruit EZH2 and LSD1 to p57 promoter regions and repress their transcription, which promoted the progression of LAD.

In summary, our study showed for the first time that FEZF1-AS1 was overexpressed in LAD tissues and cell lines and high level of FEZF1-AS1 was associated with poor prognosis. Moreover, proportional hazards method analysis revealed that high FEZF1-AS1 might be considered as a specific biomarker of poor prognosis for LAD. Influence on LAD cell proliferation, cell cycle and apoptosis suggested that FEZF1-AS1 acted as an oncogene in LAD tumorigenesis. Our findings further helped the understanding on LAD pathogenesis and facilitated the development of lncRNA-directed diagnostics and therapeutics against LAD.

## MATERIALS AND METHODS

### Human tissue samples

The 80 LAD tissues and their pair-matched noncancerous tissues described in this study were obtained from patients with advanced LAD in Department of Thoracic surgery, PLA General Hospital, Beijing March 2006 and September 2010. And all the informed consents were obtained from the patients.

### Cell lines culture and transfection

LAD cell lines (SPCA-1, NCI-H1299, A549, NCI-H441, LTEP-a2) and a normal lung epithelial cells BEAS-2B were purchased from the Tumor Cell Bank of Chinese Academy of Medical Science (Shanghai, China), and then were cultured in RPMI 1640 medium containing 10% fetal bovine serum and ampicillin and streptomycin at 37°C in a humidified atmosphere with 95% air and 5% CO_2_. Transfections were performed by using the Lipofectamine 2000 kit (Invitrogen) according to the manufacturer’s instructions.

### Real-time quantitative reverse-transcription polymerase chain reaction (qRT-PCR)

Total RNAs from tissues and cells were isolated with Trizol reagent (Invitrogen, CA, USA) according to the manufacturer’s instructions. Reverse transcription was performed with PrimeScript RT reagent Kit (Takara, Japan) according to the manufacturer’s protocol. qRT-PCR was performed with SYBR Prime Script RT-PCR Kits (Takara, Japan), based on the manufacturer’s instructions. The genes’ levels were calculated with the 2^-ΔΔCt^ method, which was normalized to GAPDH mRNA, respectively. All assays were performed in triplicate. The expression levels were relative to the fold change of the corresponding controls which were defined as 1.0. PCR primers were designed as the following: FEZF1-AS1 forward: AGAGGCTATGACTCAGGGTT, reverse: TGTTGCTCCACAGTAAAGGT; p15 forward: TCTCCGTTGGCCGGAGGTCA, reverse: TGCGCAGGTACCCTGCAACG; p16 forward: CCAGCGCATCGCGTCTC, reverse: TAGAGATCGCCGCTTGGA; p21 forward: CAGCAGACCACCATTTCA, reverse: GGTGTCTAGGTGCTCCAGGT; p27 forward: GGCTGTCTCCGCTCATAG, reverse: GCACTCTCCAGGAGGACACA; p57 forward: GGTGTCTAGGTGCTCCAGGT, reverse: GCACTCTCCAGGAGGACACA; GAPDH forward: GGGAGCCAAAAGGGTCAT, reverse: GAGTCCTTCCACGATACCAA.

### Cell viability

Cells were seeded into 96-well plates (3 × 10^3^ cells/well) directly or 24 h after transfection. After treatment with the indicated drug combinations for 48 h, cell viability was assessed via 3-(4, 5-dimethylthiazol-2-yl)-2, 5-diphenyl-trtrazolium bromide (MTT) assay.

### Colony formation assay

Cells (500 cells/ well) were plated in 6-well plates and incubated in RPMI 1640 with 10% FBS at 37°C. Two weeks later, the cells were fixed and stained with 0.1% crystal violet. The number of visible colonies was counted manually.

### Flow cytometric analysis of apoptosis

Cells transfected with indicated plasmid or negative control were reaped after 48 hours. Apoptosis assaywas performed by using flow cytometric analysis with Annexin V: FITC Apoptosis Detection Kits (BD Biosciences, USA), according to the manufacturer’s instructions. All samples were assayed in triplicate.

### Flow cytometric analysis of cell cycle distribution

Cells were collected directly or 48 hours after transfection, washed with ice-cold phosphate-buffered saline (PBS), and then fixed with 70% ethanol overnight at –20°C. Fixed cells were rehydrated in PBS for 10 minutes and incubated in RNase A (1 mg/ml) for 30 min at 37°C, then the cells were subjected to PI/RNase staining followed by flow cytometric analysis with a FACScan instrument (Becton Dickinson, Mountain View, CA) and Cell Quest software (Becton Dickinson, San Jose, CA) as described previously [26].

### Western bolt analysis and antibodies

Total protein lysates were separated in 10% sodium dodecyl suifate-polyacrylamide gel electrophoresis (SDS-PAGE), and were electrophoretically transferred to polyvinylidene difluoride membranes (Roche). Protein loading was estimated by using mouse anti-GAPDH monoclonal antibody. The membranes were blotted with 10% non-fat milk in TBST for 2h at room temperature, washed and then probed with the rabbit anti-human CDK2 (1: 2000 dilution), CDK4 (1: 2000 dilution), CDK6 (1: 2000 dilution), cleaved-caspase3 (1: 2000 dilution), total-caspase3 (1: 2000 dilution), cleaved-caspase9 (1: 2000 dilution), total-caspase9 (1: 2000 dilution), cleaved-PARP (1: 2000 dilution), total-PARP (1: 2000 dilution), p57 (1: 2000 dilution) and GAPDH (1: 3000 dilution) overnight at 4°C, followed by treatment with secondary antibody conjugated to horseradish peroxidase for 2 h at room temperature. The proteins were detected by the enhanced chemiluminescence system and exposed to x-ray film. All antibodies were purchased from Abcam (USA).

### Subcellular fractionation location

The separation of nuclear and cytosolic fractions was performed by using the PARIS Kit (Life Technologies) according to the manufacturer’s instructions.

### Chromatin immunoprecipitation (ChIP)

EZ ChIP™Chromatin Immunoprecipitation Kit was used to perform chromatin immunoprecipitation (ChIP) for cell line samples (Millipore, Bedford, MA). The crosslinked chromatin DNA was sonicated into 200-to 500-bp fragments. Normal mouse IgG was used as the negative control. The antibodies for the ChIP assays were purchased from Millipore. Quantification of the immunoprecipitated DNA was performed by using qPCR with SYBR Green Mix (Takara). The ChIP data were calculated as a percentage relative to the input DNA by using the equation 2 [Input Ct- Target Ct] × 0.1 × 100.

### RNA immunoprecipitation (RIP)

Magna RIP™RNA-Binding Protein Immunoprecipitation Kit (Millipore, USA) was utilized for RNA immunoprecipitation (RIP) experiments, based on the manufacturer’s instructions. The antibodies for the RIP assays of EZH2, EED, SUZ12 and LSD1 were purchased from Abcam. The total RNAs were the input controls.

### RNA pulldown assay

FZEF-AS1 transcripts were transcribed by using T7 RNA polymerase (Ambio life) *in vitro*, then by using the RNeasy Plus Mini Kit (Qiagen) and was treated with DNase I (Qiagen). Purified RNAs were biotin-labeled with the Biotin RNA Labeling Mix (Ambio life). Positive control, negative control and Biotinylated RNAs were mixed and incubated with SPC-A1 cell lysates. Then magnetic beads were added to each binding reaction, and incubated at room temperature. Finally, the beads were washed, and the eluted proteins were detected by western blot analysis.

### Statistical analysis

The SPSS 17.0 statistical analysis software was used for the statistical analysis of the experimental data. The significance of differences between groups was estimated by Student’s *t*-test. Multiple group comparisons were analyzed with one-way ANOVA. The overall survival probability was analyzed by using Kaplan-Meier methods and evaluated by using the log-rank test. Cox proportional hazards regression model was generated to identify factors associated with overall survival through a multivariate survival analysis of LAD. Statistically significant positive correlation between p57 and FEZF1-AS1 expression levels in 80 cases of LAD tissues was analyzed by Spearman’s correlation analysis. *P* value less than 0.05 was considered significant.
